# Bacterial compositions of indigenous Lanna (Northern Thai) fermented foods and their potential functional properties

**DOI:** 10.1371/journal.pone.0242560

**Published:** 2020-11-18

**Authors:** Chonthicha Pakwan, Thararat Chitov, Panuwan Chantawannakul, Manop Manasam, Sakunnee Bovonsombut, Terd Disayathanoowat

**Affiliations:** 1 Department of Biology, Faculty of Science, Chiang Mai University, Chiang Mai, Thailand; 2 Environmental Science Research Center (ESRC), Chiang Mai University, Chiang Mai, Thailand; 3 Department of Thai Art, Faculty of Fine Art, Chiang Mai University, Chiang Mai, Thailand; 4 Research Center in Bioresources for Agriculture, Industry and Medicine, Chiang Mai University, Chiang Mai, Thailand; 5 Research Center of Microbial Diversity and Sustainable Utilization, Chiang Mai University, Chiang Mai, Thailand; Tallinn University of Technology, ESTONIA

## Abstract

Many indigenous fermented foods of Northern Thailand and neighbouring regions have traditionally been known for their health benefits. In this study, we explored the communities of bacteria in selected fermented foods which are commonly consumed among ethnic groups around Northern Thailand, for which information on their microbial compositions or their functional properties is still limited. The selected food groups included Thua Nao (alkaline fermented soybean product), Nham (fermented pork sausage/loaf), Nam phak (fermented Chinese cabbage) and Miang (fermented leaves from Miang Tea trees). Bacteria in these fermented foods were isolated and enumerated. Bacterial communities were determined using a culture-independent (pyrosequencing) approach. Lactic acid bacteria were recovered from all of these fermented food samples, with levels ranging from 3.1 to 7.5 log CFU/g throughout the fermentation processes. Analysis of the *16S rRNA* gene from the fermented food samples using 454-pyrosequencing resulted in 113,844 sequences after quality evaluation. Lactic acid bacteria were found in high proportions in Nham, Nam phak and Miang. *Bacillus* was predominant in Thua nao, in which significant proportions of Lactic acid bacteria of the family *Leuconostocaceae* were also found. Groups of lactic acid bacteria found varied among different food samples, but three genera were predominant: *Lactococcus*, *Lactobacillus* and *Leuconostoc*, of which many members are recognised as probiotics. The results showed that these traditional Thai fermented food products are rich sources of beneficial bacteria and can potentially be functional/probiotic foods.

## Introduction

Many raw materials, such as fish, meats and vegetables have been used to make various traditional fermented products throughout the world. In Thailand, there are indigenous products such as Nham (fermented pork sausage or fermented meatloaf), Miang (fermented tea leaves from Miang Tea trees), Nam phak (fermented Chinese cabbage) and Thua nao (alkaline fermented soybean product). These products, although have some common features with other fermented foods of similar types in South China and South East Asia, are unique to Thai culture, particularly Lanna or Northern Thai culture. However, some of these products have also been popularly consumed and increasingly recognised as health foods in many other parts of Thailand.

**Fig 1:**
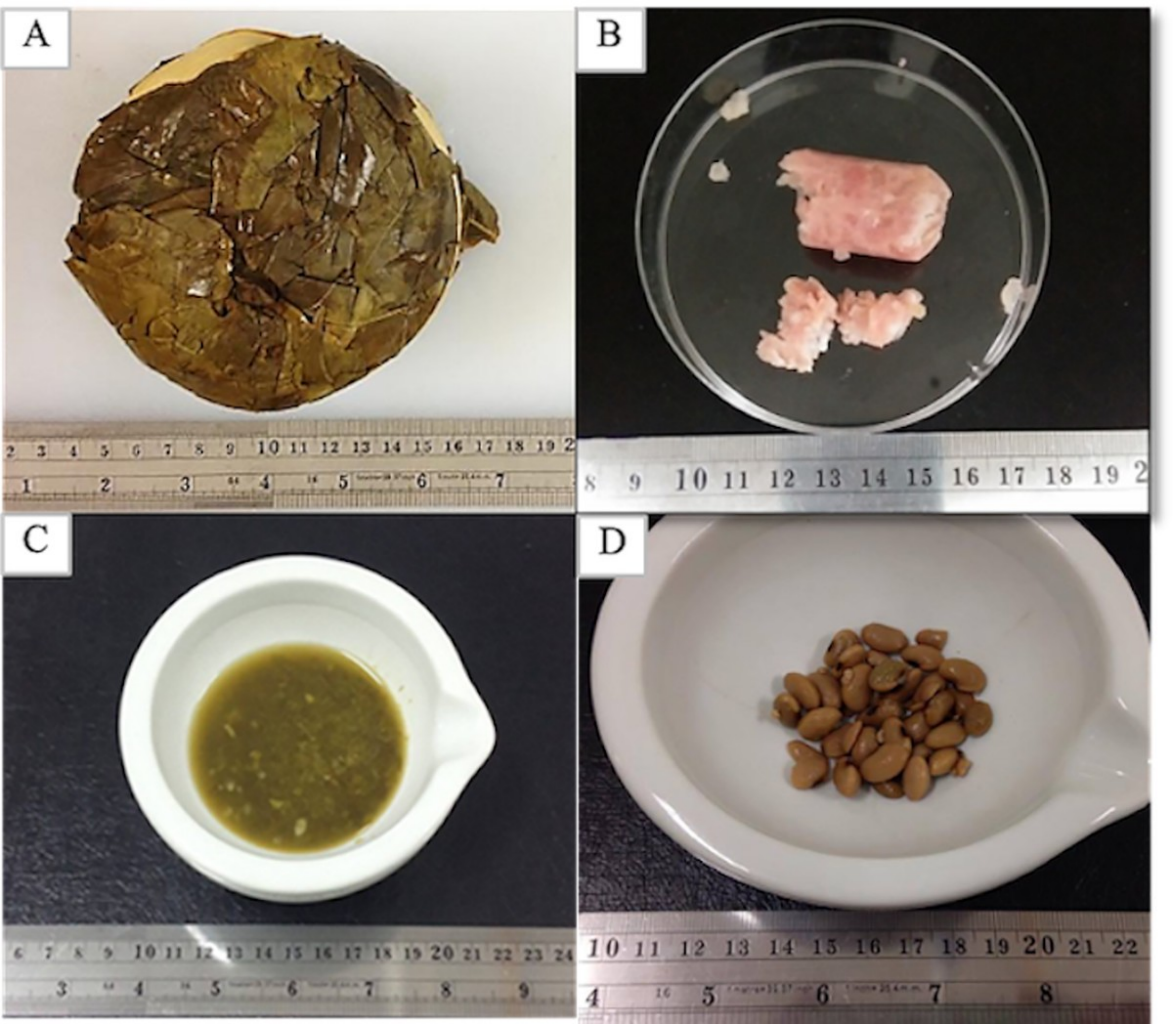
The appearance of Thai fermented products. (A) Miang (fermented tea leaves), (B) Nham (fermented pork sausage), (C) Nam phak (fermented Chinese cabbage) and (D) Thua nao (fermented soybeans).

Most of the traditional Thai fermented products are manufactured using raw materials available locally or, in some cases, raw materials indigenous to the geographical area and natural starter cultures, *i*.*e*. natural fermentation processes. A prominent example of this is Miang. This is a fermented product named after the Miang tea tree (*Camellia sinensis* var. *Assamica*) from which leaves are used for fermentation and is traditionally consumed as snacks in the northern part of Thailand [1]. To make Miang, leaves from Miang tea tree are steamed and fermented, then kept under an anaerobic condition [1]. The fermentation process takes several weeks or months allowing the sour taste and flavour to fully develop [1]. Another example: Nham is usually made of ground pork (*Sus scrofa domesticus*) [2]. It is usually made in the form of sausage or meatloaf and can be consumed uncooked or cooked. Nam phak is a fermented vegetable product made of fermented Chinese cabbage (*Brassica rapa* ssp. *chinensis*). It is usually consumed as a side dish. Nam phak is similar to Korean *kimchi*, which has the cabbage as a main ingredient. Thua nao is made from soybean (*Glycine max* (L.) Merrill) [3]. It is usually used as an ingredient in savory dishes and is available in a wet or dry form. In its dry form, Thua nao can be extensively preserved for several months [4]. The appearance characteristics of these traditional Thai fermented products are shown in Fig 1.

Beneficial microorganisms, especially lactic acid bacteria (LAB), are involved in the fermentation process of Miang, Nham and Nam phak [5–7]. LAB that are commonly isolated from Miang include *Lactobacillus plantarum*, *Lactobacillus camelliae*, *Lactobacillus thailandensis* and *Pediococcus siamensis* [5,8]. In Nham, predominant microorganisms were found to be *Pediococcus* spp., *Lactobacillus* spp. and *Micrococcus* spp. [9,10]. There is little information about Nam phak, but in Kimchi, a similar type of product, *Leuconostoc mesenteroides*, *Lactobacillus brevis* and *Lactobacillus plantarum* were among the predominant species reported [11]. As for Thua nao, unlike the other traditional Thai fermented products mentioned above, bacteria that play a major role in fermentation are those belonging to genus *Bacillus*, especially *B*. *subtilis* [4,12]. Several other *Bacillus* species, such as *B*. *pumilus*, *B*. *megaterium* and *B*. *licheniformis*, were also found in this or similar products [13,14]. These species are associated with the proteolytic process during soybean fermentation [4], which gives this type of product its unique flavour.

Many members of LAB and *Bacillus* groups have been recognised for their function as probiotics for humans. Because of the production of lactic acid and other antimicrobial substances, LAB can also inhibit undesirable pathogenic bacteria in the fermented products themselves, thus promoting natural preservation. In addition, some Lactobacilli can also inhibit microbial pathogens in the gastrointestinal tract. Therefore, these traditional fermented foods, which are associated with LAB and *Bacillus*, could be considered potential foods for promoting human health [15]. In fact, Miang and Thua nao, apart from being consumed as snacks and used as seasoning ingredients, have traditionally been known for their functional and therapeutic properties, on which fermenting microorganisms play a major role.

Most of the studies on the microorganisms involved in fermentation of these products or their varieties have been carried out using cultured methods. However, for traditional fermented products such as these, many types of microorganisms usually exist throughout the fermentation process and contribute to the unique characteristics of the products, although not all of them can be recovered or distinguishably isolated in the laboratory. Recently, next-generation DNA sequencing has been used to demonstrate a broader range of bacterial communities in food [16]. However, for these traditional Thai fermented food products, there is still limited data. This study, therefore, was aimed to identify bacterial taxa, with a particular focus on LAB present in Miang, Nham, Nam phak and Thua nao, using both culture-dependent and culture-independent approaches, by means of isolation and characterisation of the isolates and *16S rRNA* gene sequencing (454-pyrosequencing), respectively. The results obtained from both approaches were expected to reveal predominant bacterial taxa and a broader range of bacterial communities; some of which can potentially be probiotics and may contribute to the probiotic and functional properties of these traditional fermented foods.

## Materials and methods

### Sampling

In this study, four Northern Thai fermented foods (Miang, Nham, Nam phak) and Thua nao) were sampled from selected locations in different districts (Tambon Thepsadet in Doi Saket, Tambon Suthep in Mueang Chiang Mai, Tambon Malika in Maeaii, in Chiang Mai province and Tambon Pang Moo in Mueang Mae Hong Son, Mae Hong Son province, respectively), where the products are produced in the traditional ways. Briefly for the fermentation process of Miang, Miang tea leaves are steamed for 1–2 hours, left to cool, then packed and fermented with or without filamentous fungi and kept under an anaerobic condition [1]. Nham is generally made of ground pork with the addition of salt, cooked rice and garlic. Chili pepper is sometimes added. The mixture is fermented for 2–3 days under an anaerobic condition [2]. Nam Phak is a fermented Chinese cabbage. The cabbage is soaked in brine and left to ferment for 2–3 days. Thua nao is made from soybean, which after being soaked in water overnight and boiled for 3–4 hours, is left to ferment for 2–3 days [3]. Miang samples were collected at the beginning of the fermentation process (day 0) and after fermentation for 1 and 5 months. Nham and Nam phak samples were collected at day 0 and after fermentation for 1, 2 and 3 days. Thua nao samples were collected at day 0 and after fermentation for 1 and 2 days. The samples collected were kept in ice and immediately transported to the laboratory and kept refrigerated (4°C) until use.

### Isolation of total bacteria, lactic acid bacteria (LAB) and fungi

A ten-gram portion of each sample was homogenised in 90 mL of 0.85% (w/v) sodium chloride and ten-fold serial dilutions were prepared until the desired concentrations were obtained. The suspensions from different dilutions were spread onto Tryptic Soy Agar (TSA), de Man Rogosa Sharp (MRS) agar with Bromocresol green added as a pH indicator and Yeast Extract Malt Extract (YM) agar. All agar plates were incubated at 37°C for 24–48 hours. Colonies grown on TSA, MRS agar and YM agar were counted as total bacteria, presumptive lactic bacteria and fungi, respectively. The numbers of colonies were calculated into colony forming units per gram (CFU/gram) of sample.

### Biochemical identification of LAB

Presumptive LAB isolates obtained from MRS agar were analysed for their morphology, Gram-stain reaction, catalase production, CO_2_ production and protease production. The LAB colonies were preserved in 20% (v/v) glycerol at -20°C for further analysis.

### DNA extraction and PCR amplification

The fermented foods with the highest proportions of LAB were selected for high-throughput sequencing analysis. All fermented samples were homogenised in sterile distilled water and 1.8 mL portions of the food homogenates were centrifuged at 13,000 g for 3 min. Genomic DNA was extracted from the cell pellets using the MO BIO Power Food Microbial DNA isolation kit and QIAGEN DNeasy Blood and Tissue kit according to the manufacturer’s instructions. The fragments of *16S rRNA* gene were amplified by polymerase chain reaction using the universal bacterial primers 27F (5´-AGAGTTTGATCMTGGCTCAG-3´) and 1492R (5´-TACGGYTACCTTGTTACGACT-3´). A 25 μL PCR reaction mixture contained 1×buffer, 0.2 mM dNTPs, 1.5 mM MgCl_2_, 0.8 μM of each primer, 1 unit of Tag DNA polymerase (Invitrogen), 1 μL of DNA template and ddH_2_O. The PCR was carried out in a thermal cycler (BIO RAD), using the following condition: an initial denaturation for 3 min at 94°C; 30 cycles of denaturation for 45 sec at 94°C, annealing for 30 sec at 55°C and extension for 1.5 min at 72°C; and a final extension for 10 min at 72°C. The PCR products were analysed using gel electrophoresis on a 1% (w/v) agarose gel, stained with ethidium bromide and visualised under a UV transilluminator.

### 454-pyrosequencing and data analysis

The genomic DNA samples prepared as above were subjected to GS-FLX Titanium 454 pyrosequencing (Roche) (performed by Macrogen (Seoul, South Korea)). The total bacterial *16S rRNA* fragment was amplified using primers 27F (5´-GAGTTTGATCMTGGCTCAG-3´) and 518R (5´-WTTACCGCGGCTGCTGG-3´). The sequence data were processed using Quantitative Insights Into Microbial Ecology (QIIME) version 1.9.1 [17]. The low-quality sequences (<200 or >600) were removed and chimeric sequences were also discarded using USEARCH61 [18]. The retained sequences were clustered into operational taxonomic units (OTUs) as 97% sequence identity using cdhit [19], the longest sequence was picked from each OTU and the taxonomic classification was carried out using the Greengenes [20] and RDP databases [21]. Non-metric multidimentional scaling (NMDS) was calculated to collate bacterial communities associated with each of the fermented foods used in this study, using the Paleontological statistics software package (PAST) version 3.1 [22].

## Results

### Enumeration and isolation of total bacteria, lactic acid bacteria (LAB) and fungi

The numbers of total bacteria, LAB and fungi in Thai fermented foods (Miang, Nham, Nam phak and Thua nao), as recovered from Tryptic Soy Agar (TSA), de Man Rogosa Sharp with Bromocresol green (MRS-bromocresol green) and Yeast Extract Malt Extract (YM) agar, at different fermentation times are shown in Table 1.

**Table 1:** Total bacterial count, LAB and fungi in Thai fermented foods at different times of fermentation.

Fermented food	Fermentation time	Log number of microorganism *(*log CFU/g*)*		
		Total bacteria	LAB	Fungi
Miang	0 day	5.8±0.1^a^	0	0
	30 days	6.1±0.4^c^	6.4±0.3^b^	6.3±0.5^a^
	150 days	6.0±0.2^b^	6.3±0.4^a^	6.5±0.6^b^
Nham	0 day	5.0±0.1^b^	3.5±0.4^a^	n.d.
	1 days	5.0±0.2^b^	4.2±0.2^b^	n.d.
	2 days	5.5±0.4^c^	5.3±0.7^c^	n.d.
	3 days	4.8±0.4^a^	5.2±0.4^c^	n.d.
Nam phak	0 day	5.2±0.4^a^	3.1±0.1^a^	n.d.
	1 days	7.5±0.6^c^	7.5±0.5^c^	n.d.
	2 days	7.4±0.6^c^	7.4±0.3^c^	n.d.
	3 days	7.2±0.2^b^	7.2±0.2^b^	n.d.
Thua nao	0 day	4.1±0.4^a^	0	4.3±0.4^a^
	1 day	6.4±0.3^b^	6.1±0.2^a^	6.4±0.4^b^
	2 days	6.3±0.4^b^	6.2±0.5^a^	6.4±0.4^b^

Different letters in the same column (a, b, c and d) represent the differences in log numbers of microorganisms (log CFU/g) among the experiment sets, analysed using Complete Randomised Design; CRD (p≤0.05). n.d. = not detected.

From Table 1, LAB (isolated on MRS agar and tested as Gram-positive and catalase-negative) were found in all fermented food products tested. Differences between the numbers of total bacteria and LAB were particularly observed at the beginning of Miang and Thua nao fermentation. At the later stages of fermentation, LAB seemed to form the majority or a large part of the bacterial populations. The numbers of LAB in Miang at 30 days of fermentation reached the highest level and remained constant even at 150 days of fermentation (6.4 and 6.3 log CFU/g, at 30 and 150 days of fermentation, respectively). In Miang and Thua nao, besides LAB, fungal counts were also high.

In Nham and Nam Phak, LAB were detected at the beginning of fermentation, with the numbers around 3 log CFU/g. In Nham, the number of LAB reached the highest level, *ca*. 5 log CFU/g after 2 days of fermentation, which was in accordance with the gradual decrease of pH from 6.24 to 3.96 (S1 Fig). In Nam Phak, LAB increased more dramatically and reached the level of *ca*. 7 log CFU/g after 1 day of fermentation; which was the highest level of LAB among the fermented products tested. LAB remained at high levels in the later stages of fermentation for all products.

### Analysis of bacterial communities using 454 pyrosequencing

The abundance of bacterial taxa in the traditional fermented foods was investigated based on *16S rRNA* (V3-V4) sequences. From all of the fermented foods samples, 152,805 sequences were detected. A total number of 113,844 sequences were retained after discarding low-quality sequences. Based on the 97% identity reads, OTUs were generated and subjected to taxonomic classification.

**Fig 2:**
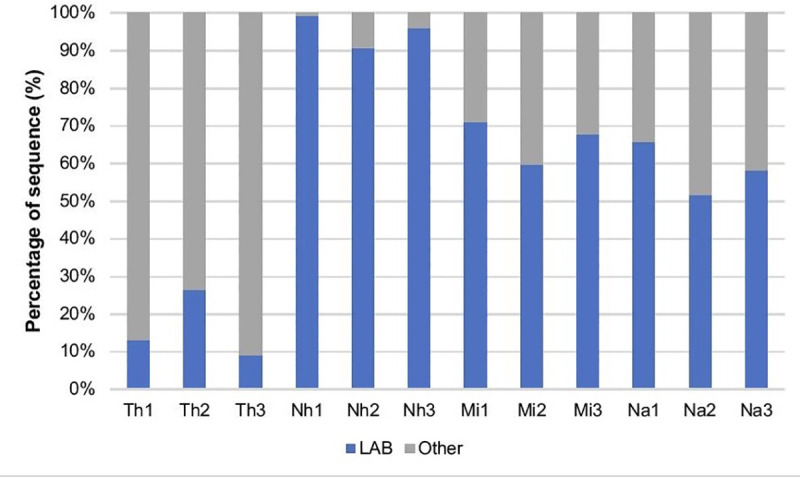
Proportions of lactic acid bacteria *(*LAB*)* and other bacteria (Other) in traditional Thai fermented foods. The data was obtained from bacterial *16S rRNA* amplicons by 454-pyrosequencing (Th-Thuanao, Nh-Nham, Mi-Miang, Na-Nam phak).

Among the traditional fermented Thai foods analysed in this study, LAB were most abundant in Nham (with the proportion of 87.12% or higher) (Fig 2). In Nham, Miang and Nam Phak, LAB group was predominant, while in Thua nao, LAB were present but the majority of microorganisms belonged to other groups of bacteria (Fig 2).

**Fig 3:**
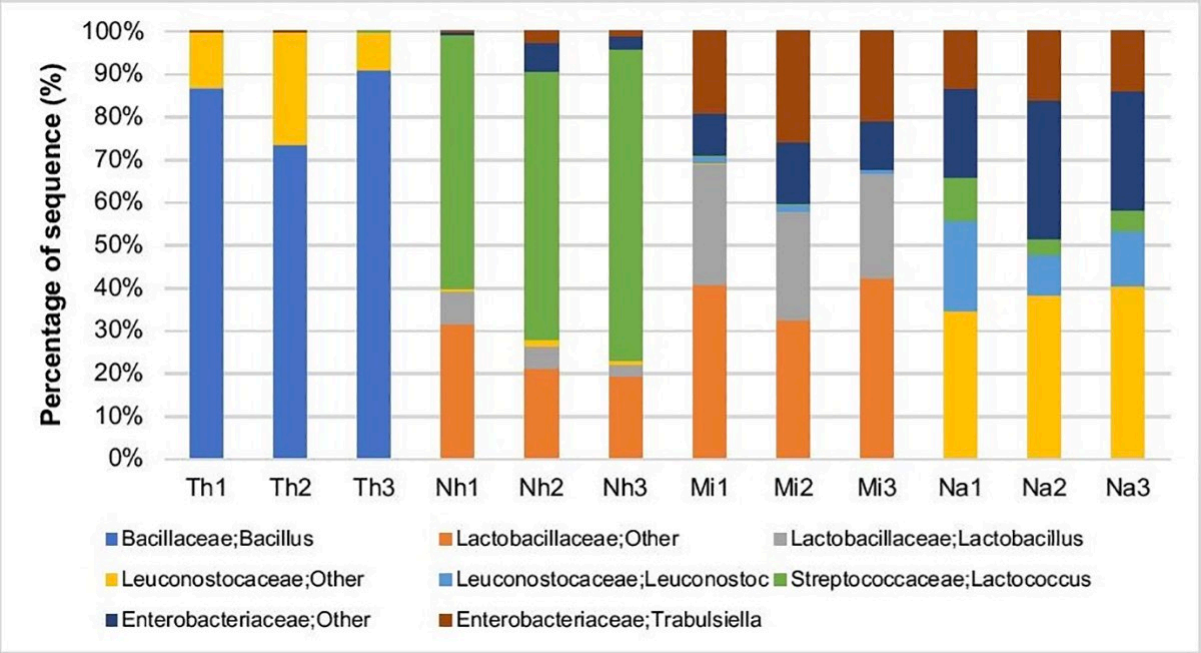
Bacterial communities in traditional Thai fermented foods (Thua nao, Nham, Nam phak and Miang). The data was obtained from bacterial *16S rRNA* amplicons by 454-pyrosequencing **(**Th-Thuanao, Nh-Nham, Mi-Miang, Na-Nam phak**)**.

A further analysis showed that, in the lactic acid fermentation class of products, the majority of LAB were members of three genera: *Lactococcus*, *Lactobacillus* and *Leuconostoc*. The majority of LAB in Nham belonged to *Streptococcaceae* and *Lactobacillaceae* families (Fig 3). Within the family *Streptococcaceae*, interestingly, *Lactococcus* was found to be the predominant genus. In Miang, the most abundant LAB group was the *Lactobacillaceae* family, while the *Leuconostocaceae* family was most abundant in Nam phak, in which genus *Leuconostoc* was found in significant proportion (Fig 3). In Thua nao, although the most abundant group was *Bacillaceae* family, especially genus *Bacillus*, *Leuconostocaceae* family was also detected (as seen in Fig 3). This confirmed the results obtained from the cultured method, from which LAB were isolated and was found in significant numbers in this product.

**Fig 4:**
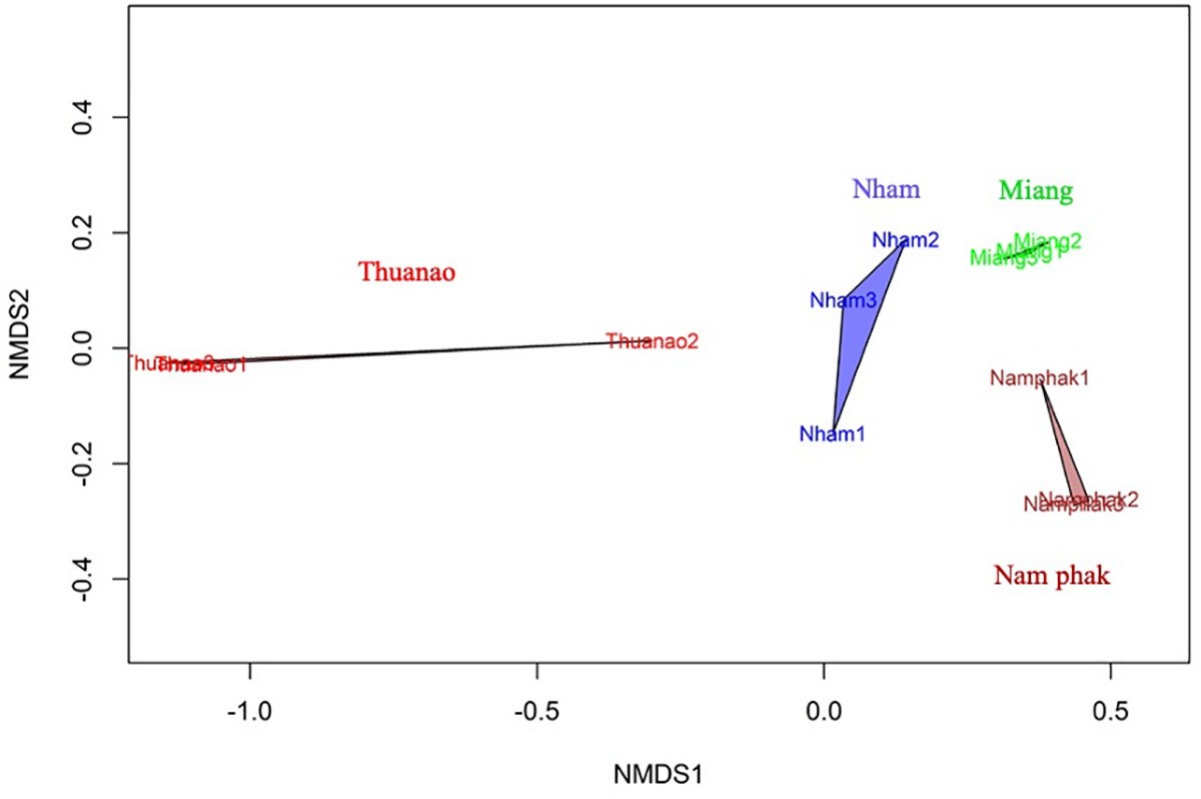
NMDS ordination plot of bacterial communities in fermented foods, with a stress value = 0.0251.

In order to analyse the similarities of bacterial communities among the different types of fermented foods, an NMDS ordination plot was used. NMDS ordination depiction of bacterial communities among the traditional fermented food samples were completely clustered away from each other, with a stress value of 0.0251 (Fig 4), indicating that each fermented product had unique bacterial communities.

## Discussion

LAB was found in every traditional fermented food used in this study. It is interesting to observe that in Thua nao, which would generally be considered an alkaline fermentation product, LAB were also present at high levels (6.1–6.2 log CFU/g) similar to those of the lactic-fermentation types of food. Such high numbers of lactic acid bacteria were previously reported in alkaline-fermented foods such as in African alkaline products [23] and in Tofu (fermented soybeans) [24]. These results suggest that some alkaline-fermented foods have a suitable environment that can accommodate LAB. Some LAB are able to grow in both acidic or alkaline media which is the result of their ability to regulate cytoplasmic pH [25]. Other studies revealed that LAB played an important role in contributing to flavour formation in Pixian bean paste [26], dairy fermentations [27], cocoa beans fermentation [28], cheeses [29,30] and juices [31]. The study of Chettri and Tamang [32] showed that LAB possessed the ability to degrade phytic acid, which is contained in plant seeds, and oligosaccharides in Indian fermented soybean foods. Phytic acid has been known for impairing mineral absorption [33]. Thus, LAB, although not the main group of organisms present in Thua nao or similar fermented soybean products, may contribute to their functional values as being a microbial agent useful for anti-mineral deficiency or for contributing to the taste and flavour of them. To fully understand this, the association of LAB with flavour in Thua nao needs to be investigated in further studies.

In Miang and Thua nao, fungal counts were high, especially in the last stages of fermentation. Although some filamentous fungi or mycotoxic fungi could be contaminants [34], many fungi are desirable and are involved in the fermentation process of various fermented soybean products, such as *Miso* in Japan, *Sufu* in China and *Tempeh* in Indonesia [35]. In Miang and Thua nao, fungi were not suppressed, even at the late fermentation phases.

In Nham, the number of LAB decreased gradually after reaching the peak at 2 days of fermentation. For Miang, the numbers of LAB after five months of fermentation slightly decreased from those after one month of fermentation. This observation was in accordance with a previous study that reported the change in microbial population during the traditional fermentation processes of fermented tea (Miang) [36].

From the analysis of bacterial communities, the bacterial group that was predominant in Thua nao was *Bacillus* spp. This finding was in accordance with other studies, which reported that the bacteria in the genus *Bacillus* played the most crucial role in soybean fermentation [4,12,14]. Various kinds of fermented soybeans can be found in Asian cultures, such as *Kinema* of India, *Natto* of Japan and *Chungkok-jang* of Korea, in which *B*. *subtilis* plays an important role in fermentation [37].

In Nham, *Lactococcus* was found as a predominant genus. This had also been observed by other researchers [38,39]. *Lactococcus* species that had been identified were *Lc*. *garvieae* and *Lc*. *lactis* [7]. Interestingly, Noonpakdee *et al*. [40] showed that *Lactococcus lactis* strain WNC 20, which originated from Nham, had inhibitory effects against some foodborne pathogens. Moreover, this strain could produce bacteriocin and was recommended for use in fermented meat products. As for Lactobacilli, the other major LAB group found in Nham in our study, some of its members are known for their potential probiotic properties [41]. *Leuconostoc* has been found among fermented foods similar to Nam phak, such as *kimchi* (fermented Chinese cabbage of Korea) [42] and *paocai* (pickled cabbages of China) [43]. In addition, *Lactobacillus* and *Weissella* were also found in the former product and *Lactobacillus*, while *Enterococcus* and *Lactococcus* were found in the latter [42,43]. Another study showed that *Lactobacillus plantarum* was associated with Miang samples collected from many provinces in Northern Thailand [44].

Some members of LAB are recognised as probiotics, which are beneficial to human’s health, particularly through the ability to survive and colonise gastrointestinal tracts of humans [45]. Some genera found in the fermented foods in this study, especially *Lactococcus*, *Lactobacillus* and *Leuconostoc*, have been known to have probiotic potential [46,47] and had positive effects on health. For example, Some *Lactobacillus* species have been reported to have cholesterol lowering properties [48] and reduce risks of microbial infection such as in the case of *Helicobacter pylori* [49,50]. *Lactococcus* species have been described for therapeutic treatments for various diseases, e.g. atopic dermatitis and inflammatory bowel diseases (IBDs) [51,52]. *Leuconostoc* sp. was reported to have an inhibitory effect against pathogens [53].

It can be seen that the traditional fermented foods, such as those from Thai culture as presented in this study, which have both economic and cultural values, can be natural carriers or sources of beneficial LAB or potential probiotic bacteria. This fact makes them potential probiotic or functional foods and the beneficial bacteria contained in these traditional food products also have great potential health benefits.

## Conclusions

This study demonstrated that lactic acid bacteria (LAB) were associated with traditional fermented foods in Lanna or Northern Thailand region. The numbers of LAB increased to significantly high levels (*ca*. 5–7 log CFU/g) in the later stages of fermentation of Miang (fermented tea leaves), Nham (fermented pork sausage) and Nam Phak (fermented cabbage), which belong to the lactic acid fermentation class of fermented foods. The culture-independent pyrosequencing analysis revealed that LAB formed the majority of a large part of the bacterial populations in these traditional fermented foods. In Thua nao, an alkaline fermented soybean product, although LAB was not the most abundant group, it reached a significantly high level at the later stage of fermentation also. Although the presence of LAB was a common characteristic in these fermented products, each of these, however, had unique bacterial communities. Many members of LAB genera found in these products are well-known as probiotics. Some members of the genera have the capacity to produce substances that contribute to products’ unique flavour, and some have potential nutraceutical or pharmaceutical properties. The indigenous foods of Northern Thailand are potential sources of beneficial LAB and could be utilised for diverse applications.

## Supporting information

S1 Fig pH of Nham during the fermentation period. (TIF)
