# Supplementary figures and images for: Bacterial compositions of indigenous Lanna (Northern Thai) fermented foods and their potential functional properties
Source: PLoS One. 2020 Nov 18;15(11):e0242560. doi: 10.1371/journal.pone.0242560 (PMC7673563; doi:10.1371/journal.pone.0242560)

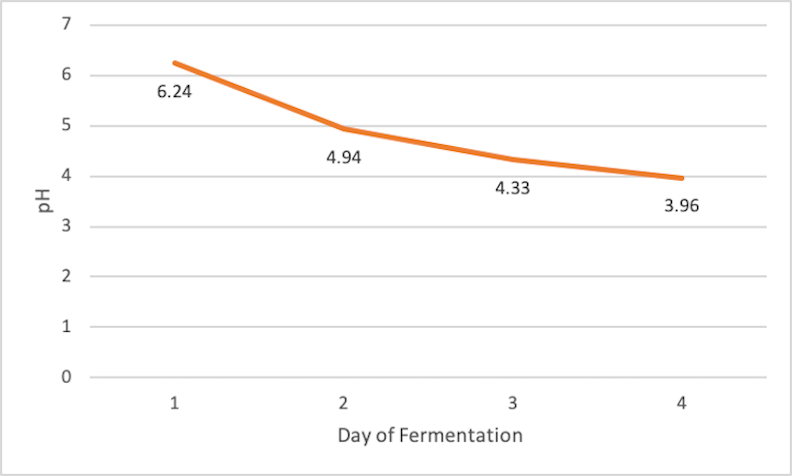

Supplement: S1 Fig — (TIF) [file pone.0242560.s001.tif]
